# Sex differences in treatment strategy for coronary artery aneurysms: Insights from the international Coronary Artery Aneurysm Registry

**DOI:** 10.1007/s12471-021-01649-5

**Published:** 2021-12-15

**Authors:** F. Arslan, I. J. Núñez-Gil, R. Rodríguez-Olivares, E. Cerrato, M. Bollati, L. Nombela-Franco, B. Terol, E. Alfonso-Rodríguez, S. J. Camacho Freire, P. A. Villablanca, I. J. Amat Santos, J. M. De la Torre Hernández, I. Pascual, C. Liebetrau, M. Alkhouli, A. Fernández-Ortiz, Coronary Artery Aneurysm Registry (CAAR) investigators

**Affiliations:** 1 Department of Cardiology, Vivantes Klinikum Am Urban, Berlin, Germany; 2 Cardiovascular Institute, Hospital Clínico San Carlos, Madrid, Spain; 3 Faculty of Medicine, Universidad Complutense de Madrid, Madrid, Spain; 4 Quirónsalud Campo de Gibraltar, Los Barrios, Spain; 5 Infermi Hospital, Turin, Italy; 6 Policlinico San Donato, Milan, Italy; 7 Hospital Severo Ochoa, Leganés, Spain; 8 Instituto de Cardiología y Cirugía Cardiovascular, La Habana, Cuba; 9 Hospital Juan Ramón Jiménez, Huelva, Spain; 10 Henry Ford Hospital, Detroit, MI USA; 11 CIBERCV, Cardiology Department, University Clinic Hospital, Valladolid, Spain; 12 Hospital Universitario Marques de Valdecilla, Santander, Spain; 13 Hospital Central de Asturias, Oviedo, Spain; 14 Department of Cardiology, Kerckhoff Heart Centre, Bad Nauheim, Germany; 15 West Virginia University Heart and Vascular Institute, Morgantown, WV USA; 16 Centro Nacional de Investigaciones Cardiovasculares, Madrid, Spain

**Keywords:** Sex, Coronary artery aneurysms, Dual antiplatelet therapy

## Abstract

**Introduction:**

Sex disparities exist in coronary artery disease (CAD) in terms of risk profile, clinical management and outcome. It is unclear if differences are also present in coronary aneurysms, a rare variant of CAD.

**Methods:**

Patients were selected from the international Coronary Artery Aneurysm Registry (CAAR; ClinicalTrials.gov: NCT02563626), and differences between groups were analysed according to sex. The CAAR database is a prospective multicentre registry of 1565 patients with coronary aneurysms (336 females). Kaplan-Meier method was used for event-free survival analysis for death, major adverse cardiac events (MACE: composite endpoint of death, heart failure and acute coronary syndrome) and bleeding.

**Results:**

Female patients were older, were more often hypertensive and less frequently smoker. They were treated conservatively more often compared to male patients and received significantly less frequently aspirin (92% vs 88%, *p* = 0.002) or dual antiplatelet therapy (DAPT) (67% vs 58%, *p* = 0.001) at discharge. Median DAPT duration was also shorter (3 vs 9 months, *p* = 0.001). Kaplan-Meier analysis revealed no sex differences in death, MACE or bleeding during a median follow-up duration of 37 months, although male patients did experience acute coronary syndrome (ACS) more often during follow-up (15% vs 10%, *p* = 0.015).

**Conclusions:**

These CAAR findings showed a comparable high-risk cardiovascular risk profile for both sexes. Female patients were treated conservatively more often and received DAPT less often at discharge, with a shorter DAPT duration. ACS was more prevalent among male patients; however, overall clinical outcome was not different between male and female patients during follow-up.

## What’s new?

- Coronary aneurysms are a manifestation of advanced stages of atherosclerosis in both men and women.
- In this study, female patients were treated conservatively more often and less frequently received aspirin or dual antiplatelet therapy (DAPT) at discharge. In addition, DAPT duration in female patients was also shorter.
- Male patients experienced acute coronary syndrome (ACS) more frequently during long-term follow-up; however, all-cause death and major adverse cardiac events (composite of all-cause death, ACS and heart failure) did not differ between the sexes.
- Despite female patients less frequently receiving DAPT and with a shorter duration, bleeding events during follow-up were similar between the sexes.

## Introduction

Underlying mechanisms and clinical outcome of coronary artery disease (CAD) are different for men and women. Studies have suggested that women exhibit less extensive CAD and less ischaemia in stable ischaemic heart disease and more frequent plaque erosion in sudden cardiac death than men [1, 2]. Nevertheless, poor clinical outcome and suboptimal management in especially postmenopausal women is of great concern. A rare but not uncommon variant of CAD are coronary artery aneurysms. The prevalence varies according to the clinical setting and geography, ranging from 0.1 to 3.5%, being around 1.4% in post-mortem studies [3, 4]. Current American and European guidelines on CAD do not mention coronary aneurysms, while their impact on clinical outcome is significant and their clinical management is very challenging due to anatomical features and lack of evidence from randomised trials [5–8].

The international Coronary Artery Aneurysm Registry (CAAR) is the first study assessing contemporary therapeutic management and clinical outcome of coronary aneurysms [9]. Recent analysis showed that the prevalence of coronary aneurysms is 0.35%. Patients with coronary aneurysms represent a very high cardiovascular risk burden with a high proportion of chronic kidney disease and peripheral artery disease. Most patients receive dual antiplatelet therapy (DAPT) or—to a lesser extent—anticoagulation and percutaneous revascularisation appear to be safe [4]. The aim of this study is to explore any differences between male and female patients with coronary aneurysms in presentation, therapeutic management and clinical outcome.

## Methods

### Study population

The rationale and design of the CAAR (ClinicalTrials.gov registration nr. NCT02563626) have been described previously [9]. In short, the CAAR is an international multicentre ambispective registry that recruited patients from 32 hospitals across nine countries (Canada, Cuba, Czech Republic, Germany, Italy, the Netherlands, Spain, United States and Uruguay).

Patients (age ≥ 18 years) were included in the registry when they met the following diagnostic criterion: a focal coronary dilation 1.5 times the diameter of a normal adjacent segment or largest coronary vessel. A giant aneurysm was defined as a 4-fold focal dilation compared with the reference vessel diameter or a focal diameter size > 8 mm. Excluded was coronary ectasias, defined as a diffuse (more than one-third of vessel length) 1.5-fold dilation of the coronary artery. Coronary aneurysm shapes were described as saccular when bulging of the vessel wall occurred on one side of the vessel and as fusiform in case of circumferential bulging on all sides.

Aortopathy was defined as a medical history of one or more of the following conditions: aneurysm, dissection or coarctation. Connective tissue disorders included scleroderma, Marfan syndrome and Ehlers-Danlos syndrome. Angiographic data were reviewed by two independent interventional cardiologists of the including centre. If no agreement was reached in the assessment of these data (e.g. eligibility, type of aneurysm or size), angiographic images were submitted to the core centre (Hospital Clínico San Carlos).

Data collection was in accordance with regulations set forth by institutional review boards and complied with the Declaration of Helsinki.

### Clinical outcome and follow-up

Clinical characteristics and complications during the index hospitalisation (e.g. cardiogenic shock, bleeding and death) were recorded. Follow-up data and events were prospectively obtained based on outpatient medical visits and records or telephone interviews. After discharge, death from any cause, hospitalisation due to unstable angina, (re)infarction, heart failure, bleeding, stroke, embolic events and any reason for coronary angiography were recorded. A combined clinical endpoint of major adverse cardiac events (MACE) was defined as the combination of all-cause death, hospitalisation for heart failure and acute coronary syndrome (ACS). The use of antiplatelet and anticoagulation therapy and the choice for revascularisation (if any) were studied during the index hospitalisation and follow-up.

### Statistical analysis

Continuous data are presented as mean with standard deviation (SD) or median with interquartile range (IQR) and categorical data as numbers (percentages of total). Differences between male and female patients were analysed using the Student’s *t*-test for continuous variables and the χ^2^ test or Fisher’s exact test for categorical variables. Kaplan-Meier estimates with log-rank test were used for survival analysis and group comparisons, respectively. The level of statistical significance was set at two-tailed *p* ≤ 0.05 (SPSS v24, IBM, USA).

## Results

A total of 1565 patients were included in the CAAR for final analysis, as described previously [4]. Compared with the male patients, the female patients (*n* = 336; 21% of total cohort) were older at presentation (65 ± 12 vs 68 ± 14 years; *p* < 0.001) and more often hypertensive (70% vs 81%; *p* < 0.001) (Tab. 1). They less frequently had a smoking habit (47% vs 21%; *p* < 0.001), less frequently had previous coronary artery disease (40% vs 27%; *p* < 0.001), less frequently had a history of aortic aneurysms or related pathology (10% vs 3%; *p* < 0.001) and less frequently had severe coronary stenosis (88% vs 82%; *p* = 0.001).

**Table 1:** Baseline characteristics

Variable	Male (*n* = 1229)	Female (*n* = 336)	*P*-value
Age, years	65 ± 12	68 ± 14	< 0.001
Diabetes mellitus	316 (26)	87 (26)	Ns
Hypertension	859 (70)	272 (81)	< 0.001
Dyslipidaemia (%)	732 (60)	197 (59)	Ns
Smoking habit (%)	575 (47)	70 (21)	< 0.001
Family history of CAD	126 (10)	34 (10)	Ns
Previous CAD	495 (40)	90 (27)	< 0.001
Peripheral vascular disease	143 (12)	32 (10)	Ns
Renal insufficiency (CrCl < 30 ml/min)	93 (8)	36 (11)	Ns
Kawasaki disease	2	2	Ns
Connective tissue disease	25 (2)	9 (3)	Ns
Aortic aneurysms or pathology	126 (10)	11 (3)	< 0.001
*Clinical presentation*			Ns
Chest pain	132 (11)	49 (15)	
Dyspnoea/heart failure	33 (3)	10 (3)	
Stable Angina	200 (16)	44 (13)	
NSTEMI	505 (41)	143 (43)	
STEMI	269 (22)	51 (15)	
Valvular study	60 (5)	35 (10)	
Severe stenosis^a^	1085 (88)	276 (82)	0.001
*Number of diseased vessels with stenosis*			Ns
1	348 (28)	89 (26)	
2	335 (27)	64 (19)	
3	402 (33)	123 (37)	
*Aneurysm type*			Ns
Fusiform	546 (44)	139 (41)	
Saccular	649 (53)	186 (55)	
Mixed	34 (3)	9 (3)	
Giant aneurysms	70 (6)	12 (4)	Ns
Size, mm (median (interquartile range))^b^	5.3 (2.5)	5.0 (1.8)	Ns
Number of aneurysms per patient (median (range))	1 (6)	1 (3)	Ns
*Coronary territory*^c^			Ns
LM	66 (5)	18 (5)	
LAD	587 (48)	175 (52)	
RCx	354 (29)	87 (26)	
RCA	408 (33)	90 (27)	
LVEF, %	54 ± 13	56 ± 13	0.027

Data are mean ± standard deviation, *n* (%) or as otherwise stated

*CAD* coronary artery disease, *CrCl* creatinine clearance, *(N)STEMI* (non‑)ST-segment elevation myocardial infarction, *LM* left main, *LAD* left anterior descending coronary artery, *RCx* ramus circumflexus, *RCA* right coronary artery, *LVEF* left ventricular ejection fraction, *ns* not significant

^a^ Severe stenosis was defined as stenosis > 70% in coronaries and > 50% in left main artery

^b^ In case of multiple aneurysms in one patient, largest size was included in analysis

^c^ More than one aneurysm could be present in same vessel and/or in different coronary territories

Diabetes mellitus, family history of CAD and dyslipidaemia were equally distributed between male and female patients. No differences were found in the clinical presentation, the extent of CAD or type of aneurysm. The size and number of aneurysms per patient did not differ between sexes, nor did the coronary territories supplied by aneurysms (Tab. 1).

Female patients received conservative treatment more frequently than the male patients (37% vs 29%, *p* = 0.01) (Tab. 2). In invasively treated patients, no differences were found in revascularised aneurysmatic segments. However, nonaneurysmatic lesions were treated more frequently in male patients (59% vs 53%; *p* = 0.042). The type of revascularisation strategy was not different between male and female patients. No sex differences were found in the choice for bare metal stents, drug-eluting stents or coronary artery bypass grafting (CABG). Female patients received significantly less frequently aspirin or DAPT at discharge. In addition, median DAPT duration was also significantly shorter compared with male patients (3 vs 9 months, *p* = 0.001). It is unknown to what extent the shorter duration was related to physician’s prescription behaviour or patient compliance. The percentage of patients on anticoagulation was higher in the female group, but this difference was not statistically significant (Tab. 2). Clopidogrel was the most frequently chosen P2Y_12_ inhibitor in both female and male patients (94% vs 93%) (data not shown).

**Table 2:** Treatment strategy at discharge

Treatment strategy	Male (*n* = 1229)	Female (*n* = 336)	*P*-value
Conservative (medical) therapy	362 (29)	124 (37)	0.01
*Revascularised segment*			
Aneurysmatic	436 (35)	125 (37)	Ns
Nonaneurysmatic	727 (59)	178 (53)	0.042
*Revascularisation strategy*			Ns
Balloon angioplasty only	40 (3)	5 (1)	
PCI – BMS	202 (16)	57 (17)	
PCI – DES	406 (33)	87 (26)	
Drug-eluting balloon	6	1	
CABG	191 (16)	59 (18)	
Aspirin	1133 (92)	294 (88)	0.002
DAPT	827 (67)	195 (58)	0.001
DAPT duration, months	9 (0–12)	3 (0–12)	0.001
Anticoagulation	163 (13)	56 (17)	Ns
Triple therapy	67 (5)	19 (6)	Ns

Data are *n* (%) or median (interquartile range)

*PCI* percutaneous coronary intervention, *BMS* bare metal stent, *DES* drug-eluting stent, *CABG* coronary artery bypass grafting, *DAPT* dual antiplatelet therapy, *ns* not significant

**Fig. 1:**
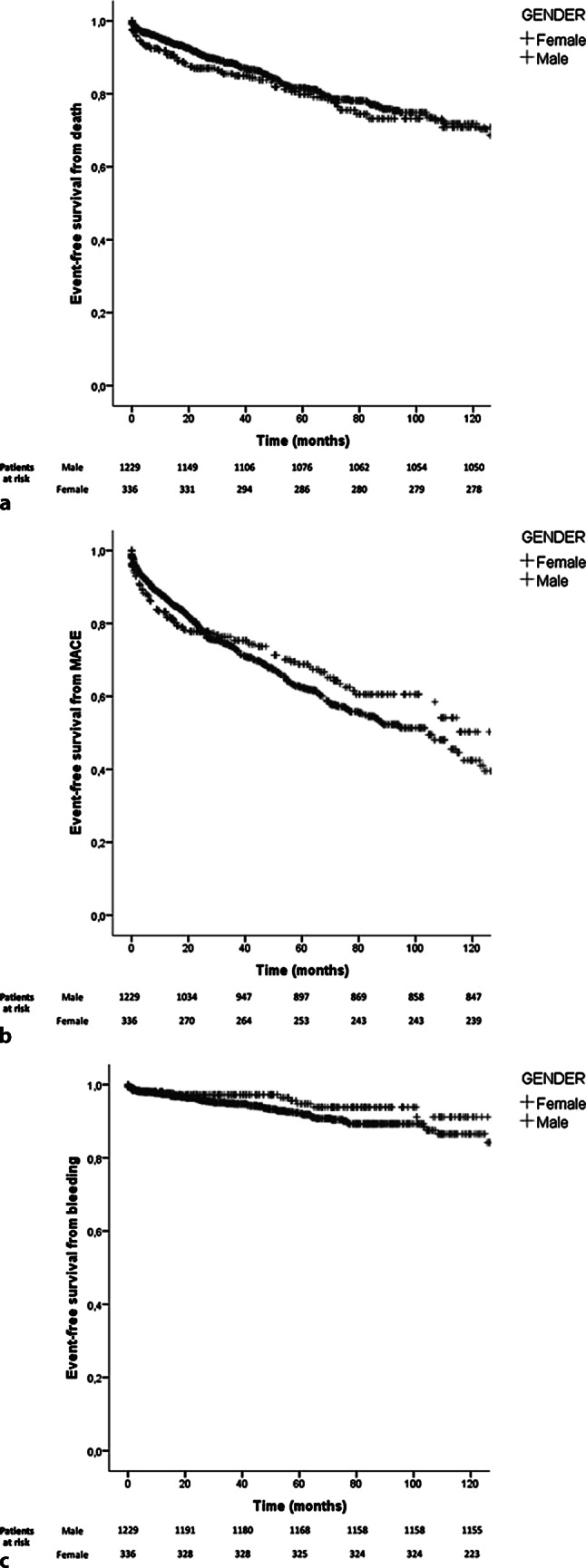
Kaplan-Meier curves for event-free survival from **a** death, **b** major adverse cardiac events (*MACE*) and **c** bleeding

Kaplan-Meier survival analysis revealed no statistically significant differences in the occurrence of death, MACE or bleeding during a median follow-up duration of 37 months (IQR 15–72) (Fig. 1). Total number of deaths was 240 (18% of females, 15% of males) (Tab. 3). MACE occurred in 485 cases (29% of females, 32% of males) and bleeding in 89 patients (4% of females, 6% of males). During follow-up, male patients were diagnosed more frequently with ACS.

**Table 3:** Outcome during follow-up

Outcome	Male (*n* = 1229)	Female (*n* = 336)	*P*-value
*Death*	181 (15)	59 (18)	Ns
Cardiovascular	57 (5)	29 (9)	0.01
*MACE*	387 (31)	98 (29)	Ns
ACS	190 (15)	35 (10)	0.015
HF	97 (8)	34 (10)	Ns
Bleeding	75 (6)	14 (4)	Ns
Stroke	25 (2)	6 (2)	Ns
Embolic events	9 (0.7)	3 (0.9)	Ns

Data are *n* (%)

*MACE* major adverse cardiac events—composite endpoint of death, acute coronary syndrome (*ACS*) and heart failure (*HF*), *ns* not significant

## Discussion

The current analysis showed that coronary aneurysms exist in the context of severe and extensive CAD and that invasive management was chosen equally often in both sexes. Surprisingly, female patients received less frequently aspirin or DAPT—and the duration of DAPT was shorter—without any differences in death, MACE or bleeding during follow-up.

Sex differences exist, and they remain challenging for ischaemic heart disease in terms of clinical presentation and management, as well as outcome. In coronary aneurysms, the challenge is even greater in the absence of guideline-supported recommendations and prospective clinical studies. In that context, the CAAR findings provide a unique insight into both clinical characteristics and therapeutic management along with long-term outcome data representative for a significant part of the world.

In the absence of other causes than atherosclerosis, coronary aneurysms are considered a variant of CAD. This is supported by the high prevalence of multiple risk factors for cardiovascular disease (Tab. 1). The subgroup analysis of the ISCHEMIA trial showed that female patients had less extensive CAD with less severe ischaemia [1]. The CAAR findings suggest that coronary aneurysms are at a different level of the CAD spectrum. The slightly higher age at presentation together with the fact that the number of vessels with severe stenosis is also high may suggest that coronary aneurysms are indeed an advanced stage of CAD.

There are many analyses showing that disparities between the sexes exist in clinical presentation and in management of ischaemic heart disease. Even in the setting of ACS, women are less likely to receive guideline-recommended therapeutics such as aspirin and statins, which may partly explain the difference in clinical outcome [10, 11].

In our study, a significantly higher percentage of female patients were treated conservatively, probably related to the lower number of severe stenotic lesions in women. Approximately 45–50% of the patients did undergo percutaneous coronary intervention and about 15% underwent CABG. Both male and female patients received any type of revascularisation to a similar degree, and both groups received mostly clopidogrel when a P2Y_12_ receptor inhibitor was prescribed. Given that equal proportions of male and female patients received invasive therapy, the differences in the use of antiplatelet therapy are surprising but are largely in line with the results of previous reports [10, 11]. Besides less frequent prescription of aspirin or DAPT in female patients at discharge, the DAPT duration was significantly shorter in female patients.

Several recent analyses from contemporary trials and registries such as PARIS, CARDIOBASE and GLOBAL LEADERS have clearly demonstrated that women have a higher risk of drug-related bleedings and that this is an important reason for DAPT cessation [12–14]. Although with the CAAR database, it was not possible to explore the arguments behind the differences in medical treatment between male and female patients, one could argue that the higher risk of bleeding complications in women may have played an important role for physicians to prescribe DAPT less often and with a shorter duration in women in the absence of clear guideline recommendations for coronary artery aneurysms. In patients with ACS, for whom guidelines clearly recommend 12 months of DAPT, the duration of DAPT did not differ between the sexes: the median duration in both sexes was 12 months. This was also the case for patients who received a drug-eluting stent: 92% of the female and 93% of the male patients who received a drug-eluting stent were given DAPT (data not shown). Again, the median DAPT duration in both sexes was 12 months. Nevertheless, the adherence to guideline recommendations for ACS was only observed for the duration of DAPT.

Additional analysis in the same ACS subpopulation revealed that the number of patients who were prescribed aspirin and DAPT at discharge were significantly lower in female than in male patients (89% vs 96%, *p* < 0.001 and 71% vs 81%, *p* = 0.032, respectively). The notion that the higher bleeding risk in women made physicians reluctant to prescribe less and shorter-lasting DAPT was also supported by the observation that bleeding events occurred to the same degree in male and female patients. Either reduced prescription of DAPT at discharge or premature cessation of DAPT during follow-up may have been related to unrecorded events of bleeding or other adverse (side) effects in female patients. Altogether, less and shorter-lasting DAPT may have reduced the bleeding rate in female patients to a level that is similar to that in male patients. In addition, the higher rate of female patients treated conservatively could also have resulted in less frequent DAPT and may be related to the lower number of severe stenotic lesions.

The issue of sex disparities is a complex interplay of psychological and biological factors, and of patients’ and healthcare providers’ perceptions on cardiovascular disease in women [15]. An important factor is the perception—of both patients and healthcare providers—that women have a low risk of cardiovascular disease, causing delays in medical contact and differences in clinical management. While the coronary anatomy was known in our study (all patients had to undergo invasive angiography) and delays in medical contact were not recorded, it might have been the perception of physicians that women with coronary aneurysms ought to be treated differently than men. It is possible that the relative rareness of coronary aneurysms caused the differences in (D)APT and its duration since there are no clear guideline recommendations or clinical trial results to guide our decision-making process. However, even in ‘clear-cut’ clinical presentations such as ST-segment elevation myocardial infarction, contemporary studies have demonstrated that women around the globe still experience differences in diagnosis, (invasive) treatment and post-myocardial infarction rehabilitation [16–18]. This demonstrates once again the importance of continuing education and increasing awareness among patients and healthcare providers.

Despite the differences in especially DAPT, there were no statistical differences in death and MACE observed between the sexes. Subgroup analysis did reveal a significant difference in the number of cardiovascular deaths (more frequently diagnosed in women). The difference in confirmed cardiovascular deaths could be related to chance or factors that are associated with observational studies, such as the impossibility to confirm the cause of death in all included patients. The higher incidence of ACS in male patients during follow-up—despite more frequent DAPT prescription—could be explained by the more than twice as high number of male smokers, more frequent previous CAD and more significant coronary stenosis.

### Study limitations

The CAAR is limited by its observational character, thereby lacking information about the rationale for shortened or altered antiplatelet regimes or the observed differences during follow-up. Despite the prospective collection of follow-up data, a systematic approach could have assured more reliable and complete data for example for drug discontinuation. A second limitation is the potential for inclusion bias because only patients who underwent invasive coronary angiography were included in the registry. This criterion skewed the population towards ACS and patients with high-risk features, while studies have clearly demonstrated that women are less likely to undergo invasive coronary angiography [10, 19]. An unknown number of patients were not included who could have been identified with other noninvasive modalities (e.g. coronary computed tomography).

## Conclusion

In the light of the current analysis, both male and female patients with coronary aneurysms have a very high-risk atherosclerotic profile, a similar extent of CAD and similar clinical outcome despite differences in antiplatelet and invasive treatment strategies. The question remains whether equality in treatment strategy would have changed the clinical outcome in female patients with coronary aneurysms. The data also suggest that we need to increase awareness regarding women’s risk of cardiovascular disease and disparities in clinical management. Future studies are necessary to clarify the impact of equal antiplatelet/antithrombotic strategy and invasive management in women with coronary aneurysms in terms of net clinical outcome (bleeding vs ischaemic risk).
